# Spheres derived from the human SN12C renal cell carcinoma cell line are enriched in tumor initiating cells

**DOI:** 10.1186/s13046-016-0442-8

**Published:** 2016-10-18

**Authors:** Yanhui Zhang, Baocun Sun, Xiulan Zhao, Huizhi Sun, Wei Cui, Zhiyong Liu, Xin Yao, Xueyi Dong

**Affiliations:** 1 Department of Pathology, Tianjin Cancer Hospital, Tianjin Medical University, Tianjin, 300060 China; 2 Department of Pathology, Tianjin Medical University, Tianjin, 300070 China; 3 Department of Pathology, Tianjin General Hospital, Tianjin Medical University, Tianjin, 300052 China; 4 Tianjin Third Central Hospital, Tianjin, 300170 China

**Keywords:** Renal cell carcinoma, Tumor initiating cells, Spheroid, Angiogenesis

## Abstract

**Background:**

Recently, tumor initiating cells (TICs), which possess self-renewal and other stem cell properties, are regarded as the cause of tumor initiation, recurrence and metastasis. The isolation and identification of TICs could help to develop novel therapeutic strategies.

**Methods:**

In this study, we isolated spheroid cells from human renal cell carcinoma (RCC) cell line SN12C in stem cell-conditioned medium. The stemness characteristics of spheroid cells, including tumorigenicity, self-renewal, proliferation and aldehyde dehydrogenase (ALDH) activity were evaluated; the expression levels of stemness genes and related proteins were assessed. Furthermore, study examined the differentiation of TICs into endothelial cells and the relationship between TICs and EMT.

**Results:**

Our data demonstrated that spheroid cells cultured in defined serum-free medium possessed TIC properties, such as high tumorigenic capacity, upregulation of TIC-related genes and proteins, persistent self-renewal and extensive proliferation. Furthermore, spheroid cells were more aggressive in growth, invasion, scratch recovery, clonogenic survival and high aldehyde dehydrogenase (ALDH) activity. Interestingly, a marked increase in tumor vascularity compared to adherent tumors in vivo, and spheroid cells can differentiate into functional endothelial-like cells in vitro suggesting a role of tumor initiating cells in tumor angiogenesis. The spheroid cells also demonstrated down-regulated E-cadherin and up-regulated Vimentin expression, which is the typical phenotype of EMT.

**Conclusions:**

These results suggest that spheroid cells with tumor initiating cells-like characteristics contributed to tumor generation, progression, high tumorigenicity, pro-angiogenic capability and relationship with EMT. Further experiments using more refined selection criteria such as a combination of two or multiple markers would be useful to specifically identify and purify TICs.

## Background

Renal cell carcinoma (RCC) is one of the commonest malignancies of the genitourinary tract accounting for 61,560 new cases and 38,270 deaths in the United States per annum [1]. Patients with RCC still face a dismal clinical outcome owing to high rate of metastasis both at initial presentation and after radical nephrectomy despite considerable improvements have been made in diagnosis, surgical techniques and adjuvant therapies in last decades [2]. Therefore, it is important to understand the molecular mechanism involved in the essence and origin of RCC. Identification of novel biomarkers associated with disease progression and metastasis of RCC and combination of their application with traditional diagnostic and prognostic parameters would contribute to development of effective strategies for the prevention, early diagnosis and treatment of RCC.

It has been assumed that tumor initiating cells (TICs) constitute a reservoir of self-sustaining cells with ability to self-renew and maintain the tumor [3–5]. A series of studies indicate that TICs may be the origin of local recurrence and distant metastases, if they were not completely eradicated by conventional treatments [6–11]. Evidence is accumulating that TICs have been isolated from several types of human tumors, such as breast cancer and brain tumors [6, 12]. However, the literature of investigating stem cells of kidney cancers is limited and lack of TIC-specific cell surface antigen markers. Currently, there are data to demonstrate that “sphere forming cells” or “spheroids” are commonly found and are useful to enrich the potential TIC subpopulations when the specific TIC makers have not been defined, as is the case for most TICs [13, 14]. Therefore, in the present study, we isolated spheroid cells from RCC cell line (SN12C) and determined whether these cells acquired TICs characteristics, including self-renewing capacity and tumorigenic capacity.

## Methods

### Cell culture

Human RCC cell line, SN12C was used for this study which was obtained from the Type Culture Collection of Chinese Academy of Sciences (Shanghai, China). SN12C were cultured in DMEM (Gibco, California, USA) medium. Both media contained 10 % fetal bovine serum (FBS, Gibco).

Spheroid cells were derived by placing in serum-free medium (SFM) consisting of DMEM/F-12 medium with 20 ng/ml EGF (Invitrogen), 20 ng/ml bFGF (Invitrogen) and B27 (Invitrogen) on poly (2-hydroxyethylmethacrylate) (poly-HEMA; Sigma-Aldrich)-precoated plates. After primary spheroid body reached the size of approximately 100–200 cells per spheroid body, the spheroid bodies were dissociated at the density of 1000 cells per milliliter and 100 single cell suspension (100 μl) was seeded in each well of a 96-well ultra-low attachment plate (Corning) in serum-free medium described above. Two weeks later, wells were analyzed for subspheroid body formation.

### Evaluation of tumorigenicity and histologic staining

All animal studies adhered to protocols approved by the local Institutional Animal Care and Use Committee. Five-week-old female BALB/C nude mice were purchased from the Animal Institute of Peking Union Medical College (PUMC). To assess tumorigenicity of spheroid cells and adherent cells, firstly, 10^4^,10^5^,10^6^,10^7^ cells of SN12C adherent cells were injected respectively into the right armpit, right inguen, left inguen and left armpit of the nude mouse(*n* = 3); 10^3^,10^4^,10^5^,10^6^ cells of SN12C spheroid cells were injected respectively into the right armpit, left armpit, left inguen and right inguen of the nude mouse(*n* = 3); secondly, 10^5^ cells of spheroid cells and adherent cells were injected s.c. respectively into the left and right inguen of the same nude mouse (*n* = 3), to comparatively evaluate their tumorigenic potential. Mice were sacrificed by cervical dislocation at a tumor diameter of 1 cm or at 6 months post-transplantation. H&E staining was done on 4 μm paraffin-embedded sections following standard protocols.

### Immunohistochemistry

Tumor specimens were fixed with buffered formalin and embedded in paraffin. Sections (5 mm) were placed on glass slides, heated at 60 °C for 20 min, and then deparaffinized with xylene and ethanol. For antigen retrieval, tumor specimens mounted on glass slides were immersed in preheated antigen retrieval solution for 20 min and allowed to cool for 20 min at room temperature. After the inactivation of endogenous peroxidase, rat anti mouse (1:50 dilution) or mouse anti human CD34 (1:200 dilution) (santa cruz) were then added, and incubated overnight at 4 °C. The second antibody was detected with a biotinylated anti-rat or anti-mouse IgG (DAKO). Diaminobenzidine tetrahydrochloride was then added for development for 10 min, followed by counterstaining with hematoxylin solution. CD34/PAS double-stained was used to validate vasculogenic mimicry (VM). It was identified by the detection of PAS-positive loops surrounding with tumor cells (not endothelial cells), with or without red blood cells in it. Microvessel density (MVD) was determined by light microscopy examination of CD34-stained sections at the “hot spot”. The fields of greatest neovascularization were identified by scanning tumor sections at low power (×100). The average vessel count of three fields (×400) was regarded as the MVD.

### Immunofluorescence

The primary antibodies were CD133-PE (1:50, Miltenyi Biotec), CD44 (1:400; Santa Cruz), CD105 (1:100; Santa Cruz), Oct4 (1:100; Santa Cruz) and Nanog (1:200; Santa Cruz). The second antibody was incubated with FITC-labeled goat anti-mouse IgG (1:200; SantaCruz). All were prepared according to the Manufacturer’s protocols. Cells were fixed with 4 % of paraformaldehyde and when needed, permeabilized with 0.05 % of Triton X-100 in PBS at room temperature for 20 min. Samples were blocked with 1 % of bovine serum albumin (Sigma) and incubated with appropriate primary antibody at 37 °C for 1 h. After washing extensively, they were incubated with second antibody at 37 °C for 1 h. After immunolabeling and washing procedure, nuclei were then counterstained with 4′6-diamidino-2-phenylindole (DAPI; Sigma). Coverslips were viewed under fluorescence microscopy (Olympus LX51, Tokyo, Japan) and photos were taken using 100-fold magnification.

### Flow cytometry

Monolayer cells were detached from culture plates with 0.25 % trypsin (Gibco), and spheroids were collected and dissociated by 5–10 min digestion with trypsin. Viable cell number was determined using Trypan blue staining (Invitrogen). Antibodies analyses were carried out as previously described [15]. The following anti-human monoclonal antibodies were used for flow cytometry: CD133-PE, CD44, CD105, Nanog, Oct4 and FITC conjugated goat anti-mouse IgG. Appropriate isotype controls were used for each antibody.

### Reverse transcription polymerase chain reaction (RT-PCR)

We examined the expression of CD44, renal stem/progenitor genes (CD133, CD105), and pluripotency gene (Oct4, Nanog) by semiquantitative reverse transcription–polymerase chain reactions (RT-PCR). The primers are provided in Table 1. Total RNA was extracted from spheroid cells and adherent cells following manufacturer’s instructions. RNA was quantified by spectrophotometry at OD260. The target cDNA was amplified using Platinum Taq DNA Polymerase (Invitrogen) for 28–30 cycles in 25 μl reactions. Aliquots of 8 μl of the amplification products were separated by electrophoresis on 1 % agarose gels and using β-actin as a loading control. Each analysis reaction was performed in duplicates or triplicates.

**Table 1:** Premier sequences and product sizes

Genes	Sense primers	Antisense primers	Products (bp)
β-Actin	5′- CGGGAAATCGTGCGTGAC -3′	5′- GAAGGAAGGCTGGAAGAGTG -3′	183 bp
CD44	5′- AATCCCTGCTACCACTTT -3′	5′- TTTCTTCATTTGGCTCCC -3′	191 bp
CD133	5′- TTACGGCACTCTTCACCT -3′	5′- TATTCCACAAGCAGCAAA -3′	172 bp
CD105	5′- TTTGGTGCCTTCCTCATCG -3′	5′- GGTTGGTGCTGCTGCTCT -3′	136 bp
Nanog	5′- CCTATGCCTGTGATTTGT -3′	5′- GTTGTTTGCCTTTGGGAC -3′	160 bp
OCT4	5′- GGTTCTATTTGGGAAGGTGTT -3′	5′- TGCTGGGCGATGTGGCTGA -3′	279 bp
CD31	5′- GCCAACTTCACCATCCAG -3′	5′- CACCCTCAGAACCTCACTTA -3′	420 bp
CD34	5′- TTGCTGCCTTCTGGGTTC -3′	5′- CATTTGATTTCTGCCTTGAT -3′	458 bp

### Western blot

The whole cell lysates were resolved by sodium dodecyl sulphatepolyacrylamide gel electrophoresis and transferred onto polyvinylidene difluoride membranes. Blots were blocked and incubated with the monoclonal antibody CD133 (1:100), CD44 (1:1000), CD105 (1:500), Oct4 (1:500), Nanog (1:1000), E-cad(1:50; CST), and Vim(1:500; Santa Cruz) and followed by incubation with a secondary antibody (1:2000, Santa Cruz, CA, USA). Blots were developed using an enhanced chemiluminescence detection kit. For protein loading analyses, a monoclonal β-actin antibody (1:2000, Santa Cruz) was used as control.

### Proliferation assay

In order to compare the proliferation rates between spheroid cells and adherent cells, 1000 spheroid cells and adherent cells were exposed to DMEM/F12 medium containing 10 % FBS, half of the medium was changed every 3 days and the number of the cells was measured and the cell growth curve was drawn accordingly. The proliferation assays were done in triplicate.

### Soft agar colony formation assay

Soft agar colony formation assay was performed by seeding cells in a layer of 0.35 % agar DMEM-F12 over a layer of 0.5 % agar/DMEM-F12. Cultures were maintained in a humidified incubator at 37 °C and 5 % CO_2_. Additional complete media was added every 2 days to continuously supply growth supplements to the cells. On day 14 or day 21 after seeding, cells were fixed with pure ethanol containing 0.05 % crystal violet and CFE quantified by light microscopy [16]. The clone formation efficiency was the ratio of the number of clones to the number of seeded cells.

### Invasion assays

Transwell chambers (8 μm pore) were first coated with 10 μg/ml fibronectin overnight at 4 °C, washed in PBS and rehydrated with serum-free media for 30 min at 37 °C. The media were removed, and then the cells (1 × 10^5^/chamber) were plated in the upper chambers of the serum-free media. The lower chambers contained media with 2.5 % FBS as a chemo attractant. Chambers were incubated at 37 °C and 5 % CO_2_. Migrated cells were stained with crystal violet solution and counted. All tests were done in duplicate.

### Wound healing (Scratch) assay

Parental and spheroid cells were cultured for 24 h. Then cells were seeded into 6-well plates to 80–90 % confluence and the cell monolayer was scratched in a straight line with a 200 μl pipette tip to create a “scratch”. Debris was removed with PBS and then the culture was re-fed with fresh medium. Images were taken at 0 and 24 h after the scratch to calculate the cell migration rate.

### Aldefluor assay by FACS

The ALDEFLUOR kit (Stem Cell Technologies, Durham, NC, USA) was used to analyze the population with a high ALDH enzymatic activity. Cells obtained from adherent or spheroid 10^9^ cells were suspended in ALDEFLUOR assay buffer containing ALDH substrate and incubated during 40 min at 37 °C. As a negative control, for each sample of cells an aliquot was treated with 50 mmol/L diethylaminobenzaldehyde (DEAB), a specific ALDH inhibitor. FACS was performed using a FACS Aria flow cytometer (BD Biosciences).

### Endothelial cell differentiation and characterization

We collected spheroid cells, washed with PBS, then added the induced medium which contained DMEM, 10 % FBS, VEGF (10 ng/mL), and bFGF (20 ng/mL). The culture were maintained for 14–21 days by induced medium and exchanged every 3 days. To examine the uptake of Dill-labeled ac-LDL (Bio-medical Technologies, Stoughton, MA), spheroid cells and differentiated endothelial cells were incubated with 10 μg/ml Dill-labeled ac-LDL for 4 h at 37 °C. After incubation, cells were washed three times with PBS, fixed with 3 % paraformaldehyde for 30 min. To investigate tube formation Assay, spheroid cells and differentiated endothelial cells (5000 cells) were seeded on the surface of the matrigel well, and were serum starved in DMEM medium for 2 h. Thereafter, they were exposed to DMEM supplemented with 5 % FBS for 24 h. Tube formation was visualized using a microscope.

### Statistical analyses

All experiments were performed at least three times and representative results were presented (quantitative data expressed as the mean ± SD). Statistical significance was set at *P* < 0.05.

## Results

### Detection of spheroid cells of SN12C

**Fig. 1:**
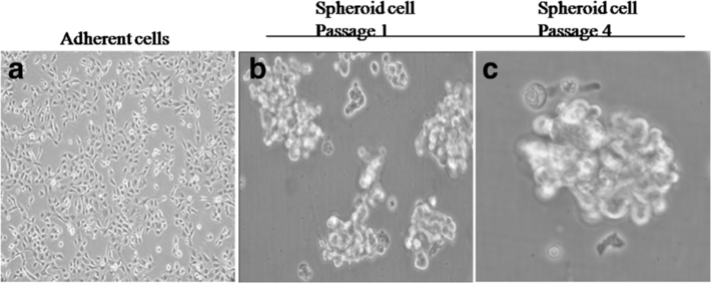
Micrograph representative of adherent cells and spheroid cells generated from SN12C cells by SFM. **a** The adherent cells. Bar = 25 μm. **b** Examples of spheroid cells isolated from SN12C adherent cells which were placed in the serum-free culture. Bar = 50 μm. **c** Spheres passaged continuously for three times. Representative staining of three independent experiments is shown

Previous studies demonstrated that stem cells were able to form spheres in the serum-free culture. We put the SN12C (Fig. 1a) into this stem cell selective condition. The floating cells aggregated into spheroid clusters after 10 days when they were placed in the serum-free DMEM-F12 supplemented with EGF, bFGF, and B27 every 48 h. Sphere-forming cells were collected 2 weeks later, each composed of 100–200 cells (Fig. 1b). The self-renewing capacity of these spheroid body-forming cells was assessed by dissociating them into single cell and growing in serum-free medium described in the methods section. SN12C subsphere can be continuously passage every 2 weeks about three or four times, without the cell shape being changed (Fig. 1c).

### Spheroid cells exhibit high tumorigenicity

**Fig. 2:**
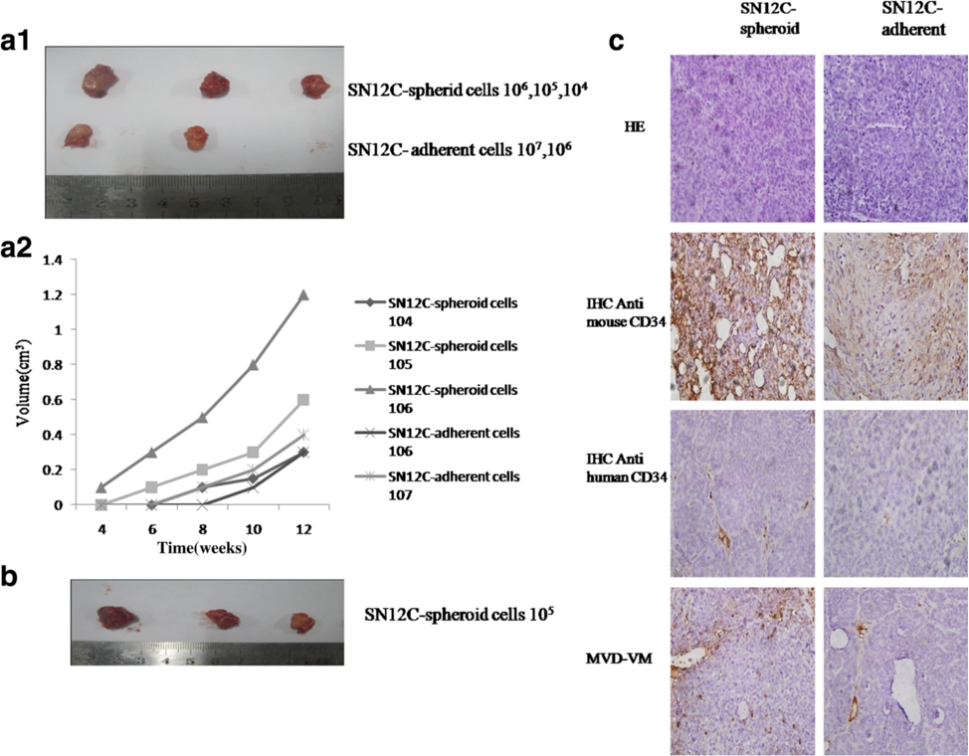
Tumorigenecity of spheroid and adherent cells in vivo. **a1** Representative xenograft tumors derived from 10^6^,10^7^ adherent cells and 10^4^,10^5^,10^6^ spheroid cells. **a2** BALB/C mice injected with different number of spheroid cells and adherent cells that developed tumors and the tumor volumes were quantified. **b** Representative xenograft tumors derived from 10^5^ cells. All mice formed tumors with spheroid cells, but not with adherent cells. **c** The H&E staining, immunostained with rat anti-mouse and mouse anti-human CD34 antibody of the tumors formed by spheroid cells and adherent cells (magnification × 100). Representative the blood microvessel density(MVD) and vasculogenic mimicry (VM) in tumors derived from spheroid cells and adherent cells was measured. The experiment has been repeated once with a similar outcome. (Magnification × 100). Representative staining of three independent experiments was shown

We examined whether smaller numbers spheroid cells (compared with adherent cells) were capable of tumorigenesis, as previously shown in other cancer TICs. Different numbers of parent, spheroid cells were inoculated into BALB/C mice to observe their ability to form tumors. At 12 weeks after cell inoculation, 10^4^,10^5^ and 10^6^ spheroid cells developed tumors in 2/3, 3/3, 3/3 mice, separately, whereas 1/3 mice injected with 10^6^adherent cells and 3/3 mice injected with 10^7^ adherent cells were tumorigenic (Fig. 2.a1). The growth rates and the average tumor volumes were significantly different (Fig. 2.a2, Table 2). We found that only 10^4^ spheroid cells could form tumor, however, more than 10^6^ adherent cells were required to form tumor in nude mice in a 3-month period. This was 100 times higher than that of spheroid body-forming cells. The tumor latency was 1–2 months by spheroid cells. As few as 10^5^ spheroid cells injected subcutaneously initiated 100 % tumor development in 4 weeks, but the same number of adherent cells was unable to form tumor in 3 months (Fig. 2b; Table 3). Hematoxylin and eosin (H&E) staining of the spheroid cell xenografts showed well differentiated tumors of typical “clear cell” morphology (Fig. 2c).

**Table 2:** In vivo tumorigenicity of different cell doses SN12C spheroid and adherent cells

SN12C	Spheroid				Adherent			
Injected cell number	10^6^	10^5^	10^4^	10^3^	10^7^	10^6^	10^5^	10^4^
Tumor incidence	3/3	3/3	2/3	0/3	3/3	1/3	0/3	0/3

Tumor incidence refers to the numbers of tumor formed/numbers of injection

**Table 3:** In vivo tumorigenicity of 10^5^ SN12C spheroid and adherent cells

SN12C	Spheroid	Adherent
Injected cell number	10^5^	10^5^
Tumor incidence	3/3	0/3

Tumor incidence refers to the numbers of tumor formed/numbers of injection

### Spheroid cells show increased angiogenesis

Our findings suggested that spheroid cells were highly more tumorigenic than the adherent cells. Microscopically, most dense tumor cells and abundant vessels were distributed in xenograft tumors derived from spheroid cells (Fig. 2c). Sometimes tumor cells seemed in contact with the lumen directly where did not show endothelium covering. Furthermore, immunohistochemical staining revealed that tumor cells which formed blood vessels tube or dispersed irregularly in the wall of tumor vessels, were stained positive to anti-human CD34, suggesting tumor cells with human origin could participate in formation of functional tumor vessels directly. Thus, increased tumor angiogenesis is a general property of SN12C-derived spheroid cells.

### Spheroid cells overexpression TIC-related genes and proteins

**Fig. 3:**
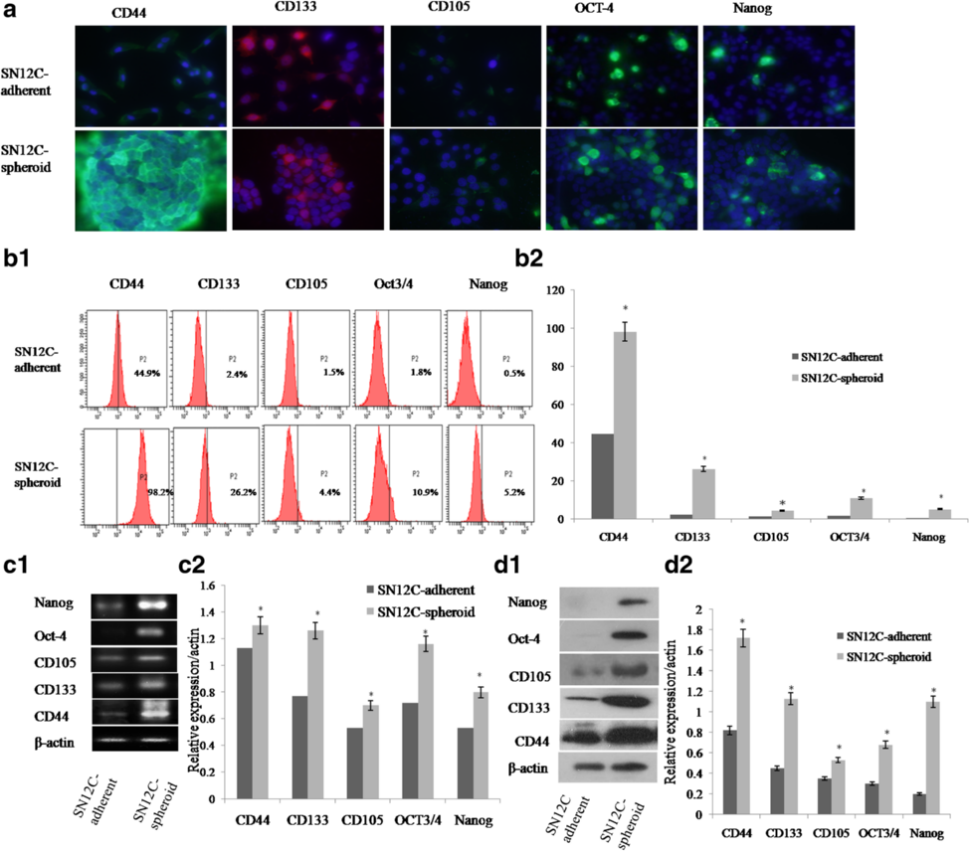
TICs-phenotype detection of spheroid and adherent cells. Representative photomicrographs depicting the higher expression of CD44, CD133, CD105, OCT-4 and Nanog in spheroid cells than adherent cells. **a** Immunofluorescence staining. Nuclei were labeled with DAPI (blue). Bar = 25 μm (adherent cells) or 100 μm (spheroid cells). **b1, b2** Representative flow cytometric analyses of spheroid cells and adherent cells stained for stem markers. **c1, c2** Semiquantitative RT-PCR was performed for analyzing expression of stem genes in the spheroid cells and adherent cells and the expression of these TICs-related genes in spheroid body cells were higher than that of adherent cells. **d1, d2** Spheroid cells were enriched for expressing stem proteins by western blot analysis. * Significant differences were detected between stem markers of spheroid cells and adherent cells. Representative staining of three independent experiments is shown

To elucidate whether spheroid cells could enrich cells expressing cell markers associated with renal progenitor cells or RCC stem cells, spheroid cells and adherent cells were stained for CD44, CD133,CD105, Oct4 and Nanog by immunofluorescent (Fig. 3a), flow cytometry (Fig. 3.b1,b2), reverse transcription PCR (RT-PCR) (Fig. 3.c1,c2) and Western blot (Fig. 3.d1,d2). All experiments showed that CD44, CD133, Oct4, Nanog and CD105 were expressed higher than adherent cells (*P* < 0.05). These data indicate that spheroid cells from RCC overexpression stem cell genes under stem cell-selective conditions and lose or 12decrease these gene expressions under differentiation conditions.

### Spheroid cells have high proliferation and migration potential

**Fig. 4:**
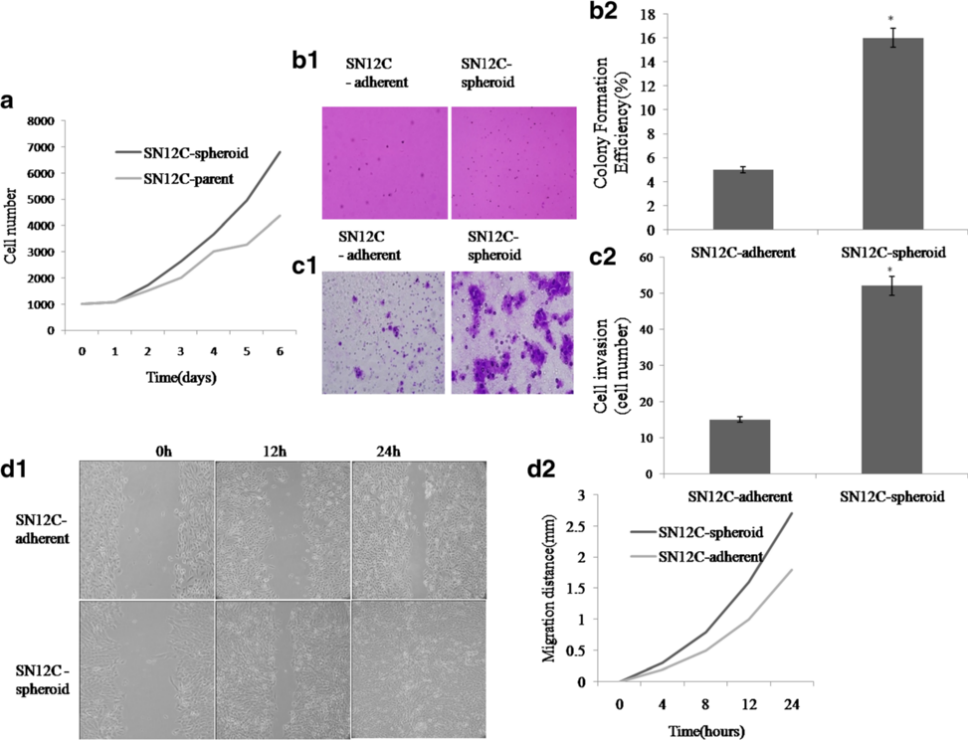
Characterization of SN12C spheroid cells and adherent cells by cell growth curve, clone formation assay, transwell assay and wound healing assay. **a** The growth curves of spheroid cells and adherent cells for 7 days. Spheroid cells proliferate at a significantly (*P* < 0.05) greater rate than adherent cells. The experiment has been repeated twice. **b1, b2** An increase clone formation in cell was observed and measured in spheroid cells when compared with the adherent cells. Bar = 25 μm. **c1, c2** The transwell invasion tests showed that spheroid cells resulted in an increased migration rate when compared with the adherent cells. Bar = 25 μm. **d1, d2** Spheroid cells recovered ‘scratch’ made by 200 μL pipette tip after 24 h more efficient than their adherent cells. * Significant differences were detected between aggressive growth patterns of spheroid cells and adherent cells. Experiments were repeated three times with similar results

We measured the proliferative and migration potential of spheroid cells and adherent cells by cell growth curve, clone formation assay, cell transwell assay and wound healing assay. The cell growth rate was calculated on the 2nd day after plating. Figure 4a showed that spheroid cells had significantly greater proliferated rate than adherent cells (*P* < 0.05). We trypsinized spheroid cells and adherent cells then subjected to soft agar colony formation assays. Our studies showed that spheroid cells exhibited higher clone forming ability and formed more colonies than adherent cells. The SN12C spheroid cells had a colony forming efficiency (CFE) of 16.4 ± 3.2 % and the adherent cells had a lower CFE of 4.9 ± 1.8 % (*P* < 0.001; Fig. 4.b1, b2). The invasion assay mimics the in vivo situation more closely. As shown in Fig. 4c, very few adherent cells invaded into matrigel while significantly more spheroid cells invaded into matrigel after 3 days of culture. Spheroid cells had higher invasion ability than their adherent cells detected on day1, 2, and 3 (Fig. 4.c1, c2). The observation that spheroid cells migrated quicker than their adherent cells was further confirmed by a wound healing assay. As shown in Fig. 4d1 and d2, more spheroid cells migrated into the scratch than adherent cells.

### Spheroid cells have high ALDH1 activity

**Fig. 5:**
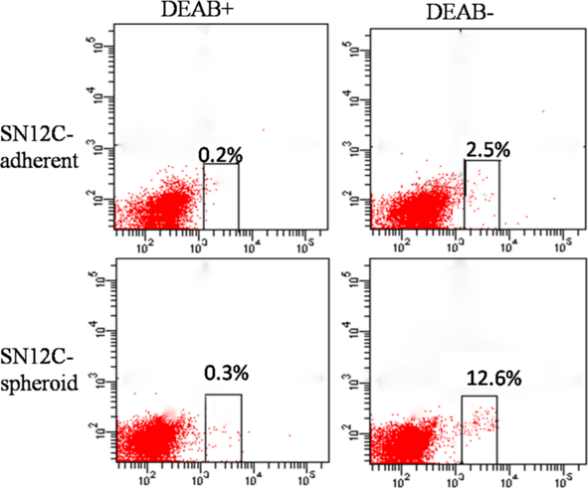
Comparative FACS analysis of ALDH-positive cells of adherent (upper, 2.5 %) and spheroid (bottom, 12.6 %) cells from SN12C

ALDH expression has been suggested as a potential functional marker for TICs. To confirm this finding, we utilized the ALDEFLUOR assay to assess the size of the population with ALDH enzymatic activity in the SN12C cell line. ALDEFLUOR-positive cells were enriched by 6 fold in spheroids, compared to the adherent cells. These results suggest that ALDH1-positive cells represent the stem/progenitor population of RCC (Fig. 5).

### Spheroid cells have upregulation EMT-associated proteins

**Fig. 6:**
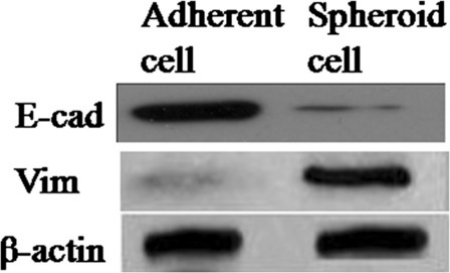
The comparative expression EMT markers in adherent cells and spheroid cells. Spheroid cells were higher expression of Vimentin, but lower expression of E-cadherin, comparing with the adherent cells

To demonstrate whether there were connections between the malignant profiles of spheroid cells and epithelial–mesenchymal transition (EMT), we detected their EMT-phenotype. The results of Western Blotting showed that Vim was higher in spheroid cells compared to adherent cells, whereas E-cadherin was lower in spheroid cells compared to adherent cells (Fig. 6). Together, these features of EMT strongly suggested a possible relationship with the TIC phenotype.

### Differentiation of spheroid cells into endothelial-like cells in vitro

**Fig. 7:**
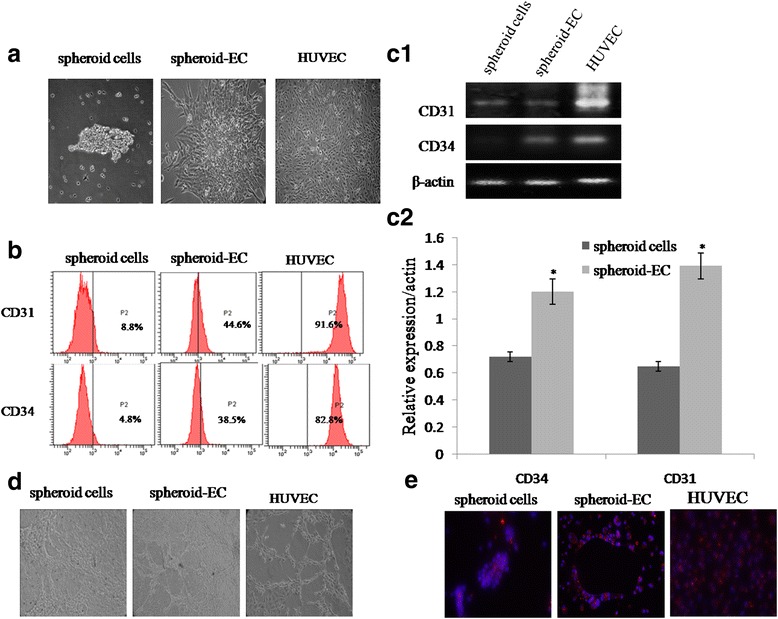
Functional analysis of spheroid-derived endothelial cells (spheroid-EC). **a** Morphological analysis of spheroid cells, spheroid-EC differentiated at 10 ng/ml VEGF, and HUVEC. **b** Flow cytometry analysis of CD31 and CD34 expression in spheroid cells, spheroid-EC, and HUVEC. **c1,c2** The comparative expression of CD31 and CD34 in spheroid cells, spheroid-EC, and HUVEC. **d** Assessment of network formation by spheroid cells, spheroid-EC, and HUVEC on Matrigel. **e** Fluorescence imaging of acetylated low-density lipoprotein (*red*) and spheroid cells, spheroid-EC, and HUVEC metabolizing ac-LDL

In order to investigate whether spheroid cells from RCC cell lines have ability to differentiate endothelial in vitro, we cultured spheroid cells in a medium containing 10 ng/ml VEGF. We observed isolated starting clones after 7 to 15 days of culture. Surprisingly, these clones, which apparently grew randomly, were easily differentiated according to their morphology, one type being cobble stone (Fig. 7a). In order to determine the phenotype of cell populations, we performed RT-PCR and flow cytometry analysis. The cobble stone population was uniformly positive for endothelial markers such as CD31 and CD34 (Fig. 7b). These results were confirmed by Western blotting analysis (Fig. 7c). In addition, these cells incorporated acetylated LDL (Fig. 7d). Taking into account their morphology, biochemical markers and functional capacity, we demonstrated that cobblestone cells were ECs.

## Discussion

Currently, there is data to demonstrate that spheroid body culture has been increasingly used as a method for enriching stem cells which relies on their property of anchorage-independent growth. Various types of potential TIC subpopulations from primary tumors have been reported to be isolated and enriched by the application of spheroid body culture [17, 18]. The spheroid body-forming cells from primary tumors, such as ovarian cancer and breast cancer, showed stem-like properties and expressed their TIC markers [14]. Since the markers of renal TICs were uncertain, it is unimaginable to isolate cells using all the markers. Also, to our knowledge, there have been few reports on the isolation and characterization of renal TICs by the method of spheroid culture, therefore, we isolated spheroid body cells by cultivating human RCC cell line SN12C in defined serum-free medium. In this report, we demonstrated that spheroid cells could generate greater numbers of new spheroid cells than the parental cells. These observations indicated that spheroid body-forming cells were capable of self-renewal and proliferation, which were important characteristics of TICs.

The gold standard assay of TICs is that the candidate population of cells can preferentially initiate tumor development in animals [19]. The least stringent definition is that the prospectively purified TICs population is much more tumorigenic than the bulk or the tumor cell population in a suitable tumor development assay. Our findings suggested that 10^4^ spheroid cells from primary tumor cells engrafted into mice and allowed full recapitulation of the original tumor, whereas 10^6^ adherent cells remained non-tumorigenic, which indicated spheroid cells were more tumorigenic than the corresponding, and the tumor formation ability of the spheroid cells overall was at least 100-fold higher than that of adherent cells. Although the adherent cells could produce tumors when large numbers of cells were implanted, the tumors that formed regressed spontaneously, whereas the spheroid-derived tumors continued to grow, indicating that the adherent cells-derived tumors may lack the TICs required sustaining tumor growth. The initial formation of tumors from high numbers of adherent cells may be explained by the high intrinsic tumorigenicity of SN12C cells, a high fraction of which express TICs markers.

To further explore the TIC properties of spheroid body-forming cells, we evaluated the spheroid cells for their stemness characteristics. Our recent work has shown overexpression of stem cell-specific transcription factor such as CD44, CD133, Oct4, CD105 and Nanog is vital characteristic of TICs [20–22]. Bussolati et al. reported the isolation of CD105+ cancer stem cells from renal carcinoma [23]. In this study, we demonstrated that CD44, CD133, Oct4, CD105 and Nanog were overexpressed in spheroid cells compared with parental cells by immunofluorescent, FCM, RT-PCR and Western blot analysis with statistical significance. So it is conceivable that the expression of CD44, CD133, Oct4, CD105 and Nanog in spheroid cells will endow some cells with certain stem/progenitor cell properties. Strikingly, spheroid cells showed approximately threefold increase in CFE when compared with that of adherent cells on soft agar, suggesting that spheroid cells have a higher self-renew potential than adherent cells. Spheroid cells showed higher migration ability than their adherent cells both in migration and wound healing assays, which were in line with Yu et al.[24], indicated that spheroid cells might promote more aggressive biological behavior. ALDHs are a superfamily of 17 intracellular enzymes that protect cells from the cytotoxic effects of peroxic aldehydes. Increased ALDH activity has also been found in stem cell populations in various types of cancer. ALDH activity may therefore provide a marker for normal and malignant stem as well as progenitor cells. Our study indicated that a high ALDH enzymatic activity is a function of RCC tumor-sphere.

Several intriguing studies have described that RCC cells developmental epithelial-to-mesenchymal transition (EMT) program to enrich their stemness features, thereby facilitating metastasis and drug resistance [25]. This is an important function, because a loss of E-cadherin on the cell surface has been shown to play a role in tumor progression and metastasis. In our study, spheroid cells demonstrated down-regulation of E-cadherin, and up-regulation of Vimentin. So, the spheroid cells display the phenotype of EMT according to our results. And the biological behaviors of spheroid cells consistent with the mesenchymal cells, such as morphological character, lower adhesion but higher in vitro migratory/invasive capacity.

Recently many accumulative evidences have showed that TICs may participate in the angiogenesis process to form endothelial cells directly [26]. It has been reported that two kinds of distinct tumor vessels play function in tumor blood-supply in malignant tumor tissues, one of which is VM, where vessels are exclusively composed of tumor cells; and the other is the endothelium vessel (EDV), where vessels are covered by the endothelium [27]. We previously also showed definite relationship between TICs, microvessel density (MVD), and VM [20]. In this study, we found spheroid cells not only formed xenograft tumors in vivo, histopathological studies have also found that some cells composing of tumor vessel lumen showed anti human CD34 positive, indicating these cells in the vessel wall of xenograft tumors were originated from the cells of human origin, not derived from host (murine) cells, which suggested that TICs might be the progenitor for tumor vasculature. This finding is consistent with recent reports that tumors grown from brain TICs, isolated based on the marker CD133, are more angiogenic than non-TIC-derived tumors [28], and that C6 glioma-derived TICs, isolated by a sphere-forming assay, exhibit increased microvessel density and blood perfusion compared with non-TIC-derived tumors [29]. Endothelial differentiation of spheroid cells were morphologically similar to HUEVC and, and displayed functional characteristics of fully mature endothelial cells. Vasculogenic potential of these cells was shown by assessing formation of networks when plated on a semi-solid substrate, and internalization of ac-LDL distinctly identified endothelial cells based on metabolic activity. These results suggest that spheroid cells are able to differentiate into functional endothelial cells in vitro and are suited for evaluation for vasculogenic potential within a tissue-engineered construct in vivo.

Together, these findings support the hypothesis that TICs promote tumor angiogenesis by secreting elevated levels of pro-angiogenic factors compared to non-TIC populations [30]. Work is in progress in our laboratory to target the specific mechanisms by which spheroid cells increased angiogenesis. Further investigation of the molecular mechanisms responsible for the increased angiogenesis seen in TIC-derived kidney tumors may improve the efficacy of cancer therapies that target angiogenesis, either alone or in combination with chemotherapy [31].

## Conclusions

In summary, we isolated RCC spheres from SN12C kidney cancer cell line and showed that they exhibit tumor initiating cells properties including high tumorigenic capacity in xenograft model, higher self-renew potential, overexpression of TICs markers, high clonogenicity and increased angiogenic potential. Further experiments using more refined selection criteria such as a combination of two or multiplemarkers would be useful to specifically identify and purify TICs.

## Abbreviations

MVD: Microvessel density
RCC: Renal cell carcinoma
TICs: Tumor initiating cells
VM: Vasculogenic mimicry
ALDH: Aldehyde dehydrogenase
